# Supplementary material for: Influence and Predictors of Anxiety on Health Status ≥ 5 Years Beyond Breast Cancer Diagnosis in Spain: A Cross-Sectional Study
Source: Life (Basel). 2025 Jun 10;15(6):932. doi: 10.3390/life15060932 (PMC12194488; doi:10.3390/life15060932)
Supplement: Supplementary file 1 [file life-15-00932-s001.zip › life-3620134-supplementary.pdf]

Supplementary Figure S1. Flow diagram for study participants

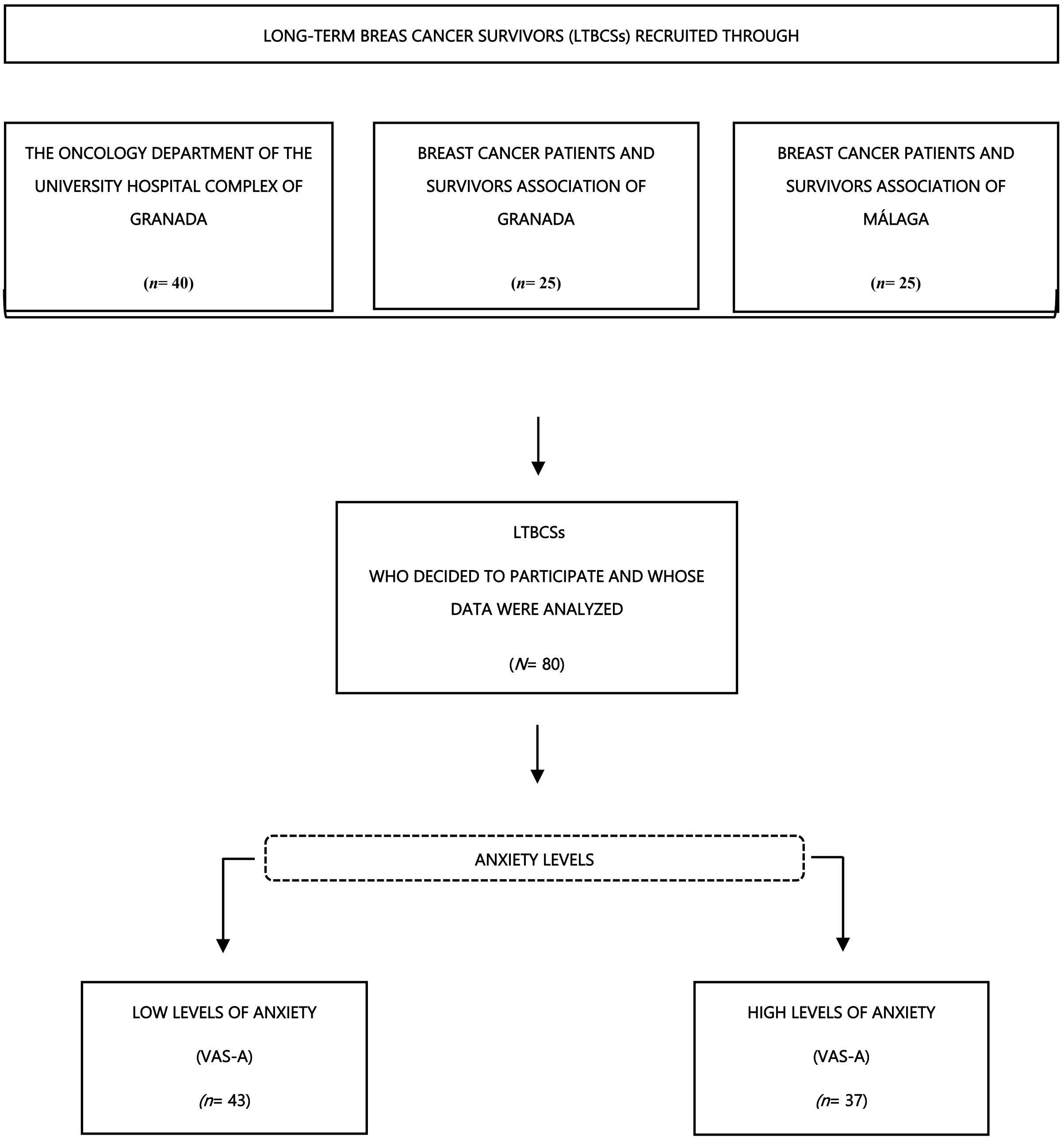

Abbreviations: LTBCSs: Long-term breast cancer survivors, VAS-A: Visual Analogue Scale for Anxiety, *N/n*: Sample size.
